# Systematic review of industry-led versus investigator-led randomized controlled trials

**DOI:** 10.1186/cc9956

**Published:** 2011-03-11

**Authors:** K Kommaraju, W Kwong, J Marshall, K Burns

**Affiliations:** 1 Virginia Commonwealth University, Richmond, VA, USA; 2 University of Toronto, Canada; 3 St Michael's Hospital, Toronto, Canada

## Introduction

Funding sources may influence the focus and conduct of randomized controlled trials (RCTs). We undertook a systematic review to examine differences between industry-funded and non-industry-funded (including mixed funding) RCTs.

## Methods

We searched MEDLINE for RCTs that enrolled at least 100 subjects and were published between 1990 and 2009 in five critical care journals (*AJRCCM*, *CCM*, *ICM*, *Chest*, *Shock*), two pediatric journals (*Pediatric CCM *and *J Pediatrics*), and five general medical journals (*NEJM*, *Lancet*, *JAMA*, *BMJ*, *Ann Intern Med*). We screened 1,094 abstracts to identify potentially eligible trials independently, and two investigators abstracted data independently and in duplicate. Statistical analysis was by the Mann-Whitney U test, *t *test or Fisher's exact test; the α level for significance was set at *P *< 0.05.

## Results

We identified 313 RCTs for which the funding source could be ascertained; 83 (26.5%) were fully industry-funded, 78 (24.9%) had mixed funding, and 152 (48.6%) received no industry funding. RCTs fully funded by industry randomized more patients (median 385 vs. 255 patients, *P *= 0.0006), used more hospital sites (63.3 ± 92.9 vs. 10.3 ± 14.8, *P *< 0.0001) and were more likely to originate from North America (51/83; 61.4% vs. 84/231; 36.4%, *P *< 0.0001). Studies investigating drugs and devices accounted for over 90% of industry-funded RCTS. Non-industry-funded trials were more likely to investigate weaning/ventilation and feeds/nutrition. A higher proportion of industry-funded RCTs recruited sepsis patients (35/83, 42.2% vs. 28/230, 12.1%, *P *< 0.0001), whereas non-industry-funded RCTs were more likely to randomize neonatal or pediatric patients (22.2% vs. 10.8%, *P *= 0.02). The number of published critical care RCTs has increased over time, from 34 from 1990 through 1994 to 116 from 2005 through 2009. The proportion of fully industry-funded trials has been constant over time. Reporting of Data and Safety Monitoring Board involvement also increased over time for both industry-funded and non-industry-funded RCTs. Studies investigating drug interventions increased over time for industry-funded RCTs, but has remained relatively constant for non-industry-funded trials.

## Conclusions

The total number of critical care RCTs has increased over time; a minority of these is fully funded by industry. Industry-funded trials are larger, more frequently originate from North America, and more frequently target patients with sepsis.

